# Modeling the Lexical Morphology of Western Handwritten Signatures

**DOI:** 10.1371/journal.pone.0123254

**Published:** 2015-04-10

**Authors:** Moises Diaz-Cabrera, Miguel A. Ferrer, Aythami Morales

**Affiliations:** Instituto Universitario para el Desarrollo Tecnológico y la Innovación en Comunicaciones, Universidad de Las Palmas de Gran Canaria, Las Palmas, 35017, Spain; University of Leicester, UNITED KINGDOM

## Abstract

A handwritten signature is the final response to a complex cognitive and neuromuscular process which is the result of the learning process. Because of the many factors involved in signing, it is possible to study the signature from many points of view: graphologists, forensic experts, neurologists and computer vision experts have all examined them. Researchers study written signatures for psychiatric, penal, health and automatic verification purposes. As a potentially useful, multi-purpose study, this paper is focused on the lexical morphology of handwritten signatures. This we understand to mean the identification, analysis, and description of the signature structures of a given signer. In this work we analyze different public datasets involving 1533 signers from different Western geographical areas. Some relevant characteristics of signature lexical morphology have been selected, examined in terms of their probability distribution functions and modeled through a General Extreme Value distribution. This study suggests some useful models for multi-disciplinary sciences which depend on handwriting signatures.

## Introduction

Learning to write is complex and usually starts with lines and scribbles. After reaching about three years of age, children begin to realize that writing is made up of lines, curves, and repeated patterns. About a year later, children begin to use letters in their own style. Usually, they start by experimenting with the letters of their own names, as they are the most familiar to them. Thus, they start to know the letters’ shapes and sequence, although the children’s motor control is not yet accurate.

Children usually start their handwriting practice using printed worksheets. These help kids to trace the letters of the alphabet and to deal with numerals. These worksheets contain writing lines that guide the height, width and length of each letter in upper and lower case and of the numbers. The tracing helps the learning of each letter shape and writing sequence. The guide lines help the spatial relationships between objects thus creating the spatial memory or cognitive map. Once this knowledge is acquired, it is possible to select an ordered sequence of target points to perform fluent writing and signatures.

At this stage, the person is ready to define and practice his or her signature. Linked to handwriting learning, the self-designed signature would depend on environmental and long term circumstances such as the signer’s personality, education, cultural environment, etc. plus the signer’s cognitive and motor skills.

Western signatures are usually written from left to right with a text line and a flourish. The flourish is a kind of random stroke, which can be curved or straight or have some kind of letter merged with it, sometimes written over the main text. It is drawn quickly and reflects more of the signer’s own personality, portraying dependencies of his or her neuromotor system’s ability and spatial cognitive map, among other factors. Also the text line design depends mainly on the signer’s name and the way the signers like to be introduced to others. For instance, let Peter Andrew Lee be a fictitious name. It could be written as P. A. Lee, Peter A. Lee and so on. The word and letter content defines the lexical part of a signature. Both parts give a particular structure or morphology to a signature.

A number of disciplines require a deeper analysis of signatures for their specific fields of interests. Neurologists, graphologists, forensic and computer scientists are actively working on this issue at different levels. Their interests in signature modeling are discussed in the following summary:

- **Biometric Recognition**: Biometric recognition takes advantage of handwritten signature variability to automatically validate personal identity. Handwriting signatures are constructed by human movement as a consequence of brain activity [1] and this process is generally stable and over-learned during growth [2]. The rapid hand movement’s velocity profiles during the signature process have been studied in depth [3–8]. Such models are currently being used for many applications such as to obtain the most relevant factors related to brain strokes [9, 10]. As a behavioral biometric, the legibility, speed, pen grip, pressure, style and error corrections are handwriting features affected by aging [11]. Experimental and practical studies have simulated aging effects [12].
- **Health**: As the signing process involves highly complex, fine motor control to generate a mostly ballistic and over-learned movement, distortion or non-usual signature variability may indicate alteration of the motor or cognitive abilities and this is important for health applications [13, 14]. Nowadays, diagnosing and preventing neurodegenerative diseases is both a medical challenge and a major concern. Patients usually perform simple handwriting tests to detect Friedreich’s ataxia [15], spinocerebellar ataxia [16] or more frequently Parkinson’s [17], Alzheimer’s [18] or Huntington’s diseases. For instance, the correlation between handwriting degradation and the grade of Alzheimer’s disease [19] is high and seems to be accepted. The effect of tremor during the handwriting process provides information about degeneration [20]. Additionally, systems able to reproduce handwritten characters from recorded electromyography signals (EMGs) have been studied [21] as a measure to assist in the diagnosis of diseases or to study statistically the neuronal variations and their correlations [22, 23]. Handwriting analysis is an additional tool for detecting disease in its early stages through clinical assessment of grip kinetics and its variation [24].
- **Graphology**: Graphology scrutinizes personality using a large set of features or symbols [25, 26]. Our signature subconsciously reflects our personality. Intra-personal variability studies generate consistent conclusions on the stability of signature features. Such features can be used, for instance, to estimate general personality, intelligence, social skill, emotions and social attitudes [27].
- **Forensics**: “Signed, sealed, and delivered” is a traditional expression for the certification of documents [28]. Contracts, testaments, corporate tax returns, government legislation or judges’ rulings are culturally accepted through a handwritten signature [29, 30]. It is crucial to validate these documents because of the many options for forgery. Forensic handwriting analysis determines the authenticity of inked or imaged signatures by a careful inspection of available samples. Other functions of a signature are made by its original owner and can be disguised [31]. Graphometric features are used in automatic signature verification [32]. These are the caliber, proportion, spacing, progression, pressure, gesture or area occupied by the features.
- **Computer Vision**: A signed document sometimes without a seal is valid to pass acceptance procedures. Nevertheless, a non-signed document or one with a forged signature could possibly be validated. Even a correctly signed document might be invalid. Legal action is often taken to resolve these matters. The issue of validation makes developments in automatic signature verifiers (ASVs) particularly important because of the variability in written signatures [33, 34].

Most of the above mentioned areas study signatures and focus on inferring a relationship between a feature space and its variability in order to establish as reliable an error margin as possible. Otherwise, the lexical morphology of the signature has been scarcely considered in the literature. In this paper we focus on the lexical morphology of Western signatures. This is understood as the identification of the most stable signature features, their analysis, and the description of the signature structures and other factors such as the presence of an decorated flourish, the number of words and letters, their distribution, the relation between them and so on.

**Fig 1:**
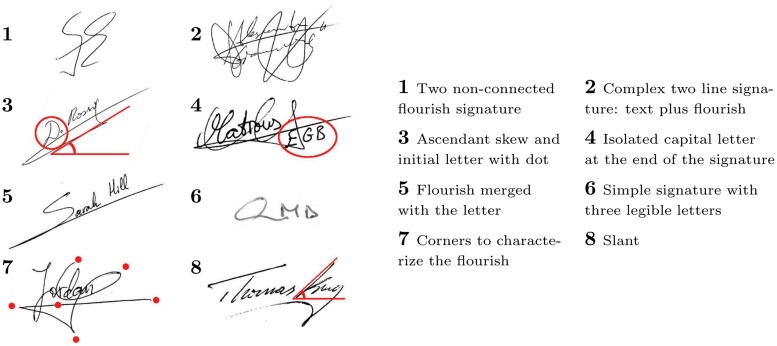
Examples of particular lexical morphological features in a set of signatures.

Such lexical morphology depends on the signer and his or her behavior and how they learned to sign. In Western signatures some particular features can be found to define the lexical morphology, for instance, signatures with one or two flourishes or no flourish; different numbers of words distributed into one, two or even three lines; capital letters sometimes followed by a full-stop; internal features such as the skew or slant; letters of different sizes against the constant size of other letters, as well as a combination of capital and non-capital letters. Fig 1 shows some of these particular and fairly common features.

The lexical morphology parameters define the signature. The more parameters we rely on, the more knowledge of the signatures we can achieve and therefore move towards a deeper understanding of the common and divergent features of the signatures for a particular culture. As a recent motivation in the biometric community, the creation of large databases with new and artificial users requires an a priori knowledge [35–37]. This is introduced through the lexical morphology distributions of real signatures to create synthetic yet credible models. As stated above, this is also useful for medical areas which study the healthy and unhealthy feature parameters in the signatures, as well as the forensic sciences, which explore the feature details among specimens. Characterizing the commonest parts of the signature implies obtaining a map of the normality of the measured parameters. All these applications require generic signature modeling.

To the best of our knowledge, the number of works analyzing the lexical morphology of signatures is few. In this paper we develop a study of the most relevant features of the Western signature lexical morphology. The identified features were statistical modeled by counting the data in several public signature databases collected in several European countries to take into account different Western signing styles. As result, a unified framework is obtained for establishing the statistical normality of a signature’s lexical morphology. This framework characterizes how the signers design their signatures and is of interest to different disciplines and applications such as forensic, graphology, indexing, etc.

## Materials and method

The set of the most relevant features that configure the lexical morphology in a signature has been analyzed from five different publicly available databases of Western signatures. These are described in the next section. The techniques we use for their statistical characterization is developed in the methodology section.

### Materials: Signature Dataset

The datasets contain collected signatures from different users. They comprise two kind of signature: *genuine/original* and *forged* signatures. *Genuine* means the signature drawn only by the owner. *Forged* refers to a signature faked or imitated from a knowledge of the genuine samples but it does not necessarily imply high forger skill.

Depending the way the signature is collected, it is called dynamic or static. A dynamic signature is captured using input devices such as special tablets with specially designed pens or PDAs. The tablet gathers the signature position coordinates and the pressure values of the pen every T seconds, usually T = 0.01 sec, with a spatial resolution of, for example, 2540 dpi. Some features extracted from these signatures can be used for expressing a person’s handwriting habit and individuality, such as pen pressure, velocity, acceleration and its direction, pen-lifts and the order of strokes. A static signature is normally drawn on paper using an inked pen and scanned after capture, often at a resolution of about 600 dpi. The ink deposition texture and the trajectory are classic features which can be extracted from the samples.

In order to address different Western styles, we have used five public databases as follows.

- The GPDS960GRAYsignature database consists of 881 users with 24 genuine signatures acquired in a single session and 30 forged signatures. In total, the database provides 881 × 24 = 21144 and 881 × 30 = 26430 genuine and forgeries signatures respectively, all scanned at 600 dpi [33]. This Spanish dataset is one of the largest off-line signature databases presented in the literature.
- The MCYT On-line and Off-line Signature database is composed of 330 users, with 25 genuine signatures acquired in two sessions and 25 forgeries. It therefore comprises 330 × 25 = 8250 genuine signatures and 330 × 25 = 8250 forgeries. The static version of MCYT gathers 75 users with the same number of repetitions for genuine and forgeries as the on-line version. This means 75 × 25 = 1875 genuine signatures (acquired in two sessions) and 75 × 25 = 1875 forged representations, all scanned at 600 dpi. Note that the captured users in the off-line version are included in the on-line database [38, 39]. This dataset has been collected in Spain and is one of the largest on-line corpuses.
- Different signature databases have emerged during past ICDAR and ICFHR conferences. The NISDCC dataset is a database used for the Signature Competition during the ICDAR 2009. It was collected and processed by the Netherlands Forensic Institute. This corpus contains the off-line and on-line versions of the same signatures. In this work we have used the evaluation corpus which comprises 100 users, with 12 signatures per writer and 6 forgeries per signature. In total, such a corpus has 1953 signatures for both on-line and off-line datasets. [40, 41].
- The two sub-corpuses from the on-line SUSIG database have also been considered. The *Visual sub-corpus* contains 94 available users, with 20 genuine signatures, acquired in two sessions, and 10 forged signatures: 94 × 20 = 1880 and 94 × 10 = 940 genuine and forged files. In total, 2820 available signatures. *The Blind sub-corpus* consists of 88 users with 8 or 10 genuine repetitions and 10 forged signatures per user. These sub-corpuses contain in total 820 genuine and 880 forged available files [42]. This database was collected at the Sabanci University in Turkey.
- The donors of the publicly available on-line SVC2004 signature database are mainly Chinese people, who are used to writing in English. This database is different to the others because there are Chinese and English style signatures included. This database is divided into two subsets: Task 1 and Task 2. Each subset contains 40 users with 20 genuine and 20 forged signatures per user. Considering both subsets, all of the databases contain 2 × 40 × 20 = 1600 and 2 × 40 × 20 = 1600 genuine signatures, captured by a multi-session protocol, and forged signatures. As this work considers only Western signatures, the Chinese signatures were omitted, leaving 40 Western users [43].

These databases were collected in countries located in western, central and eastern Europe. To study the dependence of the lexical and morphological features as a function of the donors’ geographical area, a geographical region has been assigned to each dataset. In this way, the more occidental databases, i.e. the signatures included in the GPDS and MCYT databases have been grouped. As such, we have labeled both of these databases with the name *DB1*. The nomenclature and label assigned to each dataset are shown in Table 1. As we do not know whether there is a truly Western style in the SVC dataset, such a style will be named non-native style” in this work. All experiments were performed with all databases.

**Table 1:** Database classification according their geographical area.

**Database**	**Label**	**Style**
GDPS and MCYT	DB1	West Europe
NISDCC	DB2	Central Europe
SUSIG	DB3	Eastern Europe
SVC	DB4	Non Native Style

Therefore, summing up the five datasets, the lexical morphological features have been extracted from 881 + 330 + 100 + 94 + 88 + 40 = 1533 different signers.

### Method

The method we use to characterize the lexical and morphological parameters depends on the feature properties. In this study, they are divided into three kinds: shape features (e. g. the signature envelope); discrete features (e.g. the number of words per line); and continuous features (e.g. the signature skew or slope).

In the case of the signature envelope, this is modeled by means of Point Distribution Models (PDMs) or the Active Shape Model (ASM) and consists of a mean signature shape and a number of eigenvectors to describe the main modes of variation of the shape [44].

The ASM is built as follows: Using *N* different signatures, each is converted into black and white by means of Otsu’s threshold and the salt and pepper noise is removed. Each image is morphologically dilated with a square structuring element. The envelope is the contour of the dilated signature. All the contours are aligned by moving their geometrical center to the coordinate origin. From each contour we select *n* equidistant points called landmarks so as to obtain the vector $x^{s}=\{x_{1}^{s},x_{2}^{s},\ldots ,x_{n}^{s},y_{1}^{s},y_{2}^{s},\ldots ,y_{n}^{s}\}$, where $\left(x_{i}^{s},y_{i}^{s}\right)$ are the coordinates of the *i* ^*th*^ landmark of the *s* ^*th*^ contour. The first landmark $\left(x_{1}^{s},y_{1}^{s}\right)$ is the one that satisfies $y_{1}^{s}=0$ and $x_{1}^{s}>0$. The average envelope is calculated as: $\bar{x}=1/N\sum_{i=1}^{N}x^{s}$.

The ASM captures the statistical features assuming that the point cloud *x* ^*s*^, *s* = 1,…, *N* is a 2*n* dimensional ellipsoid which is obtained by applying principal component analysis (PCA). The 2*n* × 2*n* covariance matrix is calculated as:

$$\begin{array}{c} S=\frac{1}{N}\sum_{s=1}^{N}\left(x^{s}-\bar{x}\right){\left(x^{s}-\bar{x}\right)}^{T} \end{array}$$ (1)

The principal axes of the ellipsoid are described by the eigenvectors *p*_*k*_, *k* = 1,…,2*n* of *S* and the length of its axis is related to the eigenvalues *λ*_*k*_ ≥ *λ*_*k*+1_, *k* = 1,…,2*n*.

A new envelope can be modeled using the mean shape and a weighted sum of these deviations obtained from the first *l* modes as follows:

$$\begin{array}{c} x^{f}=\bar{x}+P\cdot b \end{array}$$ (2)

where *P* = (*p*_1_,…, *p*_*l*_) and *l* is such that $\sum_{k=1}^{l}\lambda_{k}\approx 0.98\sum_{k=1}^{2n}\lambda_{k}$, and *b* = (*b*_1_,…, *b*_*l*_) is the vector of weights which are obtained randomly with a uniform distribution of mean zero and deviation equal to $|b_{k}|<4\sqrt{\lambda_{k}}$ for each vector component.

In the case of features with discrete values, the number of occurrences of each feature was manually counted for the databases to compute their occurrence probability. Each feature was validated from about 200 signatures extracted from the databases. Let $X={\left\{x_{i}\right\}}_{i=1}^{L}$ be the *L* available values of a given feature of *M* possible values. The occurrence probability of each value is worked out as *p*(*x*_*i*_) = #{*x*_*i*_ ∈ *X*}/*L*, # meaning the number of times.

In the case of features with continuous values, e.g. the skew, the values of such a feature was manually obtained using the databases and their probability density function (pdf) estimated by the histogram non-parametric method [45]. Let ${\left\{x_{i}\right\}}_{i=1}^{L}$ be the *L* available values of the given feature such that the range of this variable, range(*x*) = max(*x*) − min(*x*). This is divided into *M* intervals or bins of width *h*, which is chosen to obtain a number of intervals *M* = range(*x*)/*h* around *L*/50 to obtain a good statistical significance for each bin. The histogram is worked out as: hist(*n*) = #{*x* ∈ bin*n*} 1 ≤ *n* ≤ *M*. To generalize the estimated histogram, it is smoothed for each bin using a 3-point moving average filter as follows: shist(*n*) = PDF_*i*_ = median_*n* − 1 ≤ *l* ≤ *n*+1_{hist(*l*)}, and the density is estimated as *p*(*x*|*x* ∈ bin*n*) = shist(*n*)/*L* × *h*.

When *M* > 4, a parametric procedure is also applied to estimate a further probability density function. This parametric procedure relies on the Generalized Extreme Value (GEV) distribution [46] which is used when the feature distribution does not fit the Gaussian. These analyses could be conducted by the lognormal continuous probability distribution. However, some features such as the slant contain negative values which are not useful for lognormal. Therefore, in order to build a unique framework, a GEV distribution is proposed which has been traditionally used for modeling extremes of natural phenomena such as waves, winds, temperatures, earthquakes, floods, etc. We try to generalize the human signature variability response through a unique distribution model we use to smooth the fit according to our collected data histogram. The GEV probability density distribution has the following prescription:

$$\begin{array}{c} \frac{1}{\sigma }t{\left(x\right)}^{\xi +1}e^{-t(x)} \end{array}$$ (3)

where

$$\begin{array}{c} t\left(x\right)=\{\begin{array}{cc} {\left(1+(\frac{x-\mu }{\sigma })\xi \right)}^{-1/\xi } & \mathrm{if}\;\xi \neq 0\\ e^{-(x-\mu )/\sigma } & \mathrm{if}\;\xi =0 \end{array} \end{array}$$ (4)

with *x* bounded by *μ*+*σ*/*ξ* from above if *ξ* > 0 and from below if *ξ* < 0. The symbols *μ*, *σ* and *ξ* represent the location, scale and shape distribution parameters. The shape value determines the family of the extreme value representation from Fisher Tippett Types I, II, III which correspond to *ξ* = 0, *ξ* < 0 and *ξ* > 0 separately. Also the shape value is directly related to Gumbel, Fréchet and Weibull families according to extreme value theory.

Some of the studied parameters share common information, independently of the database analyzed. The statistical similarity of the probability density distribution of one parameter for one database comparing with the others is also analyzed. Such statistical similarity analysis is performed through two-sample Kolmogorov-Smirnov test (KS) [47–49]. This method allows us to cluster some single features from a database. For graphical representation only, we have clustered the results when the feature is statistically similar between the databases.

This non-parametric test evaluates the degree of similarity between two probability density functions. The null hypothesis *H*_0_ of the test means that two data distributions are from the same distribution. The alternative hypotheses *H*_1_ means that two data distributions are different. In our implementation, the significance level chosen is 5%. To accept the null hypothesis, the asymptotic p-value is calculated, which should be as near to 1 as possible. Such a p-value represents the probability that the null hypothesis is true by observing the extreme test under the null hypothesis.

After estimating the feature distribution parameters, the mean, variance, skewness and kurtosis values of the distribution are provided for better knowledge of the feature distribution. The mean, variance and skewness indicate the symmetry of the distribution, and the kurtosis the peakedness of the distribution. The mean square difference between the parametric and non-parametric estimation is also given.

## Results

Thousands of features can be obtained from a signature to model its lexical morphology. In this section the lexical morphological features considered most relevant, i.e. descriptive and common, are described alongside their estimated PDFs. They are presented in a top-down process, starting from global feature characterization and finishing with specific details in the signature.

### Signature envelope

**Fig 2:**
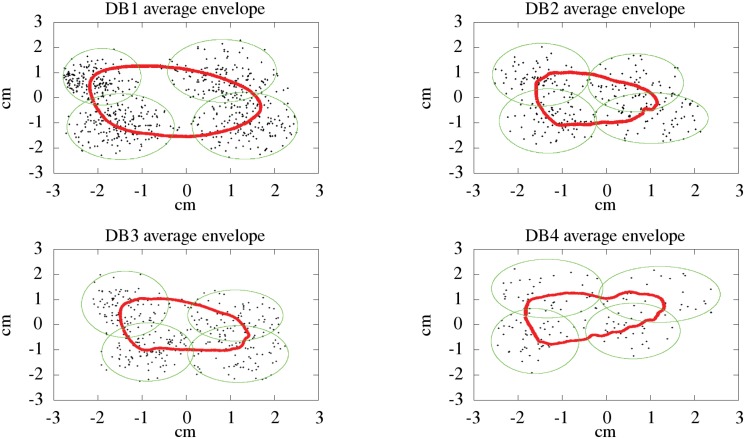
Averaged signature envelope with the cloud point around 4 landmarks out of 320.

The envelope of the signature is a fictitious shape which encloses each deposited signature. Each signature has its own specific envelope. In this study we have analyzed the average envelope of the signatures per databases by using the Active Shape Model (ASM). This method uses the images from off-line signatures to compute their contour. As DB3 and DB4 are composed of dynamic signatures, we have converted them into images by interpolating the spatial sequences and fixing the resolution at 600 dpi in all datasets. The envelope of each individual signature was smoothed through a morphological operation which was performed 3 times over each signature and also using 9 components as square structuring elements. Finally, to obtain an average signature envelope for each database, 320 equidistant landmarks were selected in this particular implementation. The average envelopes for the Western databases are shown at Fig 2. We have highlighted the ellipses of 4 equidistant landmarks for each average envelope, according to the formula (1). We can see their overall elliptical shape in all cases, which is characteristic of signatures with large text, written in a single line and with a flourish. Also we could observe how the right part of the signature is usually smaller than the left part. This is also a characteristic of Western signatures, where the initial part appears slightly bigger on average. Additionally, we can observe that the average envelope for DB1 is more rounded than the others, thus showing the stronger influence of more elaborate flourishes in this dataset.

**Fig 3:**
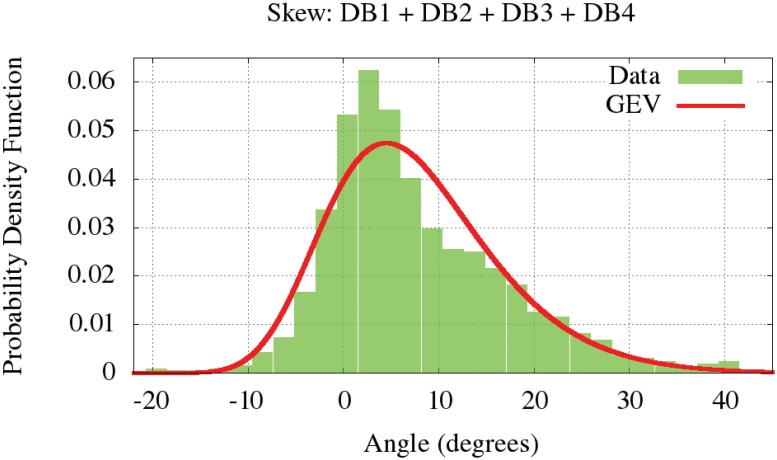
Skew PDF modeled by a GEV.

The shape of the signatures can be ascendant, descendent or longitudinal. This particular feature is measured through the skew angle, which indicates the inclination of the shape of the signature. The angle of the skew is measured in degrees and the third image in Fig 1 illustrates how it is defined. The skew distribution was calculated for the four databases. From the Kolmogorov-Smirnov approach, the skew distribution is similar for the all considered datasets, and is modeled in Fig 3. This figure indicates that the normal skew value is near to zero degrees. Also it is shown that the skew in the signatures is more often ascendant than descendent.

### Text lines morphology

Western signatures are generally composed of text, which is sometimes difficult to read because of the signing speed, plus a flourish. The text in the Western signature defines the personal identity of the signer which reflects the name, the family name or just a combination of initial letters. The flourish or rubric in the occidental signatures is defined by a kind of doodle written much faster and without much attention. It sometimes contains personal information as an almost illegible initial. Certainly, this feature is strongly dependent on the personal name of the signer. However, the analyses of this feature highlight some findings about how people decide to show their signature in different geographical areas. We could observe that in certain areas people write their full name and surname thus using a large number of letters and words in their signatures. Also we can observe that other regions prefer to use fewer letters to identify their personal signature. All of these peculiarities are analyzed in this section.

The signatures with text and flourish are the most common and are estimated to comprise 86.6% of the total of Western signatures in the DB1; 50.0% in the DB2 and; 53.5% for the DB3. However, in the DB4 the value is just 10.0%, probably because the donors are not used to signing in native Western styles. Signatures with either only a text name or only a flourish are found in proportions 5.1% and 8.3% respectively for the DB1; 33.3% and 16.7% for the DB2; 37.7% and 8.8% for the DB3 and; 90.0% and 0.0% for the DB4.

According to the learning process, people in general use their own criteria to decide the number of lines and words in their signatures. In this paper the word distribution presented in the signatures has been counted. We find that in signatures with text, the dataset DB1, 90% are written in one line whilst for the remaining 10% it is two. For the 10%, the second line is often below the first and is usually shorter than first line and starts at about 20% of the signature length toward the right side. The rest of datasets contain all signatures drawn in one line.

For those signatures written in one line, the proportion with one, two or three words is 50.0%, 36.0% and 14.0% respectively for the dataset DB1; 64.7%, 27.5% and 7.8 for DB2; 77.7% and 22.3% for DB3; and 92.5%, 7.5% for DB4. These two latter datasets do not contain signatures with three words. We are aware that the total number of words and letters per word depend on personal choice, influenced by the original name and surname. However, we note the influence of two surnames in the Spanish culture, since some signers include both.

For signatures depicted in two lines, which applies only to BD1, the proportion is as follows:

- In the first line one, two and three words appear respectively at 55.0%, 37.0% and 8.0%.
- In the second line, 71.0%, 21.0% and 8.0% of the signatures have respectively one, two and three words.

**Fig 4:**
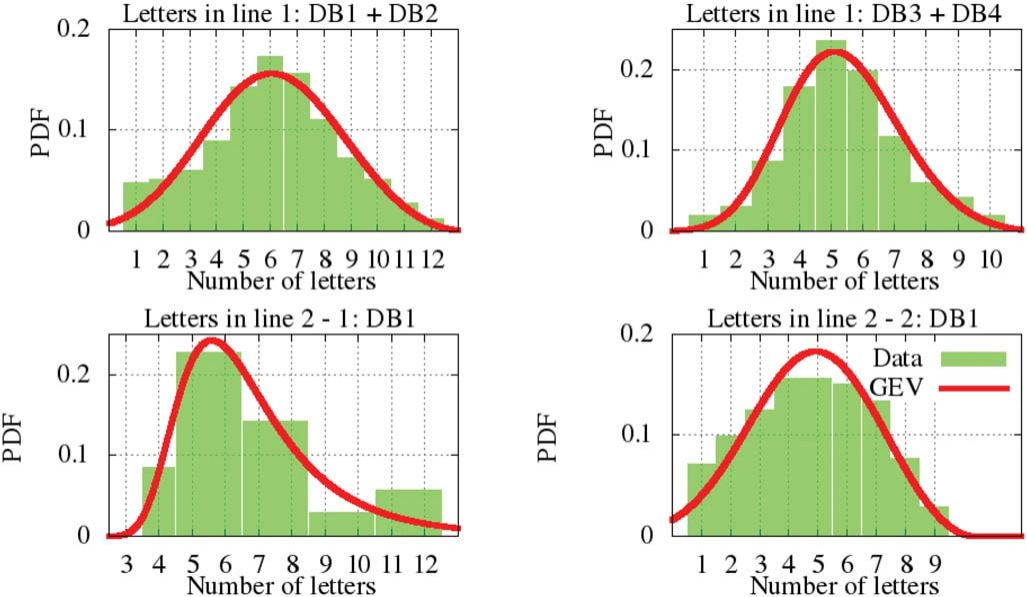
Modeling the total letters per line PDF.

For the number of letters per line, two types have been differentiated: type one is related to signatures with one line (line 1) and type two related to signatures with two lines (*line 2-1* refers to first line of a signature with two lines and *line 2-2* to the second line). Type one is found in all datasets, whereas type two is a feature only of DB1. The parametric and actual number of letter distributions are displayed at Fig 4. For the type one, the KS test suggests that DB1 and DB2 are similar among themselves. This is also true of DB3 and DB4. Such a difference was also observed during the analyses of the Western and central Western signatures because their donors usually write the full name or larger names than the other style. However, it can be seen that the model of all the datasets is 5 to 6 letters per signature, independently of the number of lines and the styles. The parameters of the parametric GEV distribution can be seen at Table 3.

**Fig 5:**
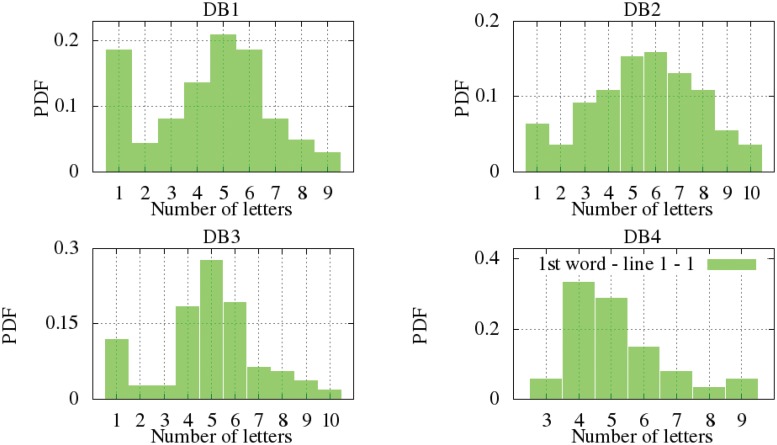
Letter distribution in the first word for signatures written in one line.

**Fig 6:**
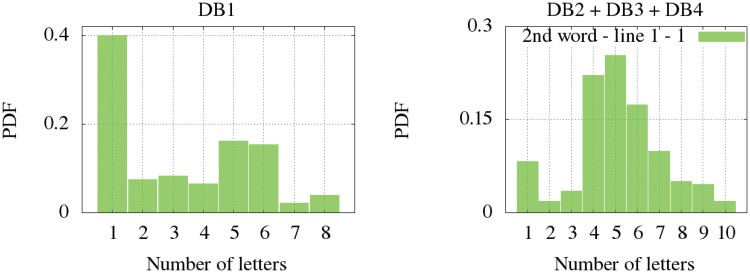
Letter distribution in the second word for signatures written in one line.

**Fig 7:**
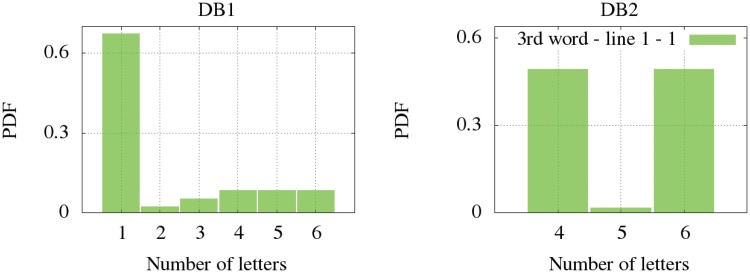
Letter distribution in the third word for signatures written in one line.

**Fig 8:**
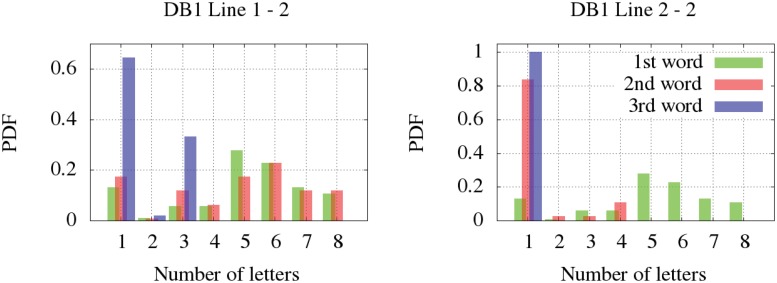
Letters per word distribution for signatures with two lines of up to three words. Left: first line of a signature. Right: second line of a signature.

Regarding the number of letters per word, we have differentiated the signatures written in one line (all databases) and the signatures written in two (DB1). Firstly, the non-parametric distribution for the signatures written in one line are shown according to the distribution of letters in the first word (Fig 5), the second (Fig 6) and the third one (Fig 7). It is observed that DB3 and DB4 are datasets whose maximum number of words is two. Once again, the Kolmogorov-Smirnov test determined the more suitable clustering representation for these feature in all databases. Secondly, the number of letters in signatures with two lines from DB1 is analyzed in Fig 8. The inferred densities present a bimodal behavior basically due to the presence of text based on names or surnames as initials whose probability is proportional to the number of words.

When the first symbol is an upper case initial, 60.0% of the signers write a full-stop after such an initial. However, these signers do not keep such behavior constant. The probability that all of a signer’s signatures retain the full-stop after the initial signing is estimated to be 73.0%. Similarly, the connectivity between letters in a word is not constant in signer behavior. We have found that, as an average, signers connect 59% of the characters in their signature.

**Fig 9:**
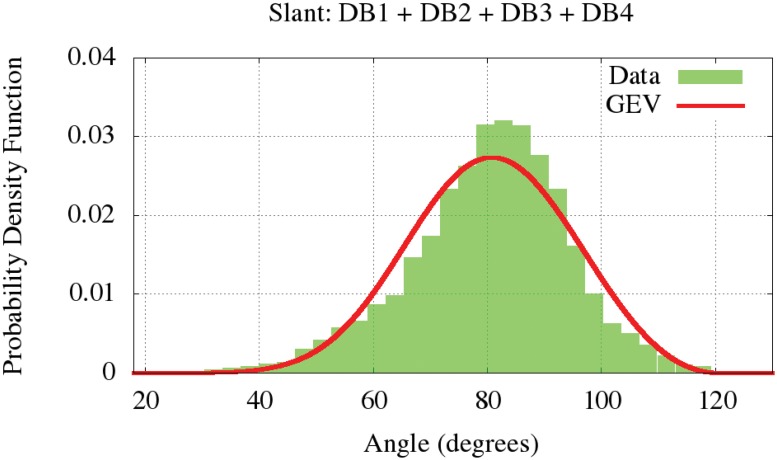
Slant model by Generalized Extreme Values (GEV).

Additionally, some people write their signatures with more rightward angle than their basic handwriting. Such an angle is called slant and it is measured in degrees. An example of how it is defined can be found in the eighth signature in Fig 1. Although the majority of text in the signatures appears fairly level, without a tilt, we perceive that there is a major tendency for right-slanted signature than left-slanted one, i.e., people tend to write in cursive style at a slight angle, away from the vertical, according to the estimated distribution in Fig 9. he statistical test estimated that the parametric distribution of slant is quite common in all databases. Such parametric values are given at Table 3.

### Flourish morphology distributions

The flourish is the part that usually introduces higher inter-personal variability in the signature. Several lexical morphological characteristics can be determined from the flourish, namely the number of flourishes and their relation with the text.

**Fig 10:**
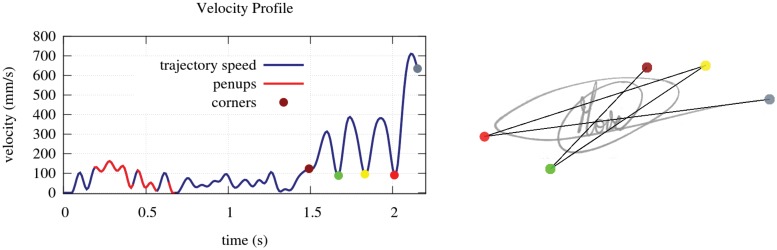
Relation between the speed profile minima and the signature “fictitious” corners.

To characterize the complexity of a flourish which is generally written quickly, we rely on the kinematic theory of rapid movements [50–53]. This theory models the trajectory as a sequence of superimposed strokes aimed at a sequence of target points. An estimation of the number of target points can be used as a measure of the flourish complexity. Similarly, we can use a complexity measure based on the number of minima in the speed profile of the flourish. This corresponds to zones where the flourish changes direction with high curvature; consequently, the signer slows down the writing. This can be said to correspond to “fictitious” flourish corners, as represented at Fig 10. The number of speed minima is smaller than the number of target points because successive strokes are superimposed.

**Fig 11:**
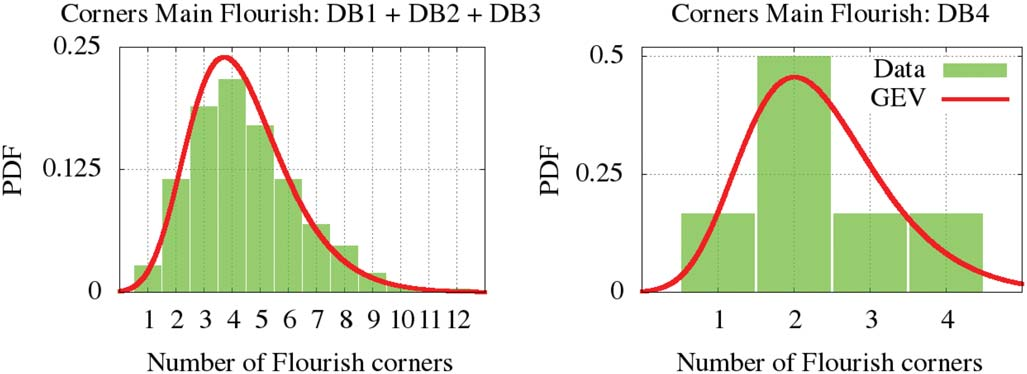
GEV modeling the corners distribution for the main flourish.

**Fig 12:**
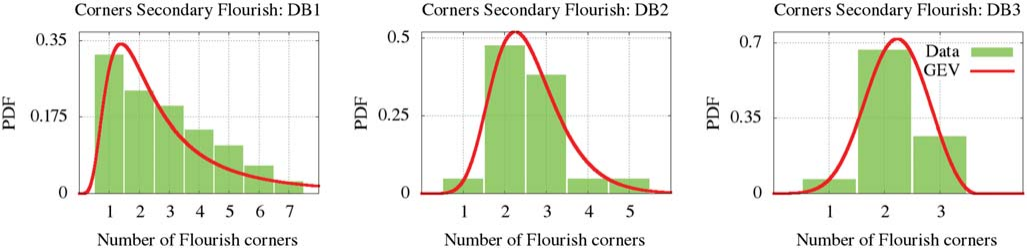
GEV modeling the corners distribution for the secondary flourish.

Some signatures are found with two flourishes. The flourishes can be distinguished as the *main flourish* (Fm), which have more “fictitious” corners and means the most elaborate one, and the *secondary flourish* (Fs), the simpler one with fewer “fictitious” corners. The estimated parametric and non-parametric distributions of the number of corners for the main flourishes (Fm) and secondary flourishes (Fs) are represented in Figs 11 and 12. According to the statistical similarity given by the KS test, we can observe in the plots that the number of corners of the first flourish is similar in the first, second and third databases. We found that the elaboration of the secondary flourish is more notable in the DB1 and DB2. We can also deduce that the signers decorate their second flourishes with fewer corners than the main one. The parameters of the GEV are provided at Table 3 for all these cases. DB4 has few signatures with a flourish, which were only just worthy of analysis.

### Some text-Flourish morphology dependencies

**Fig 13:**
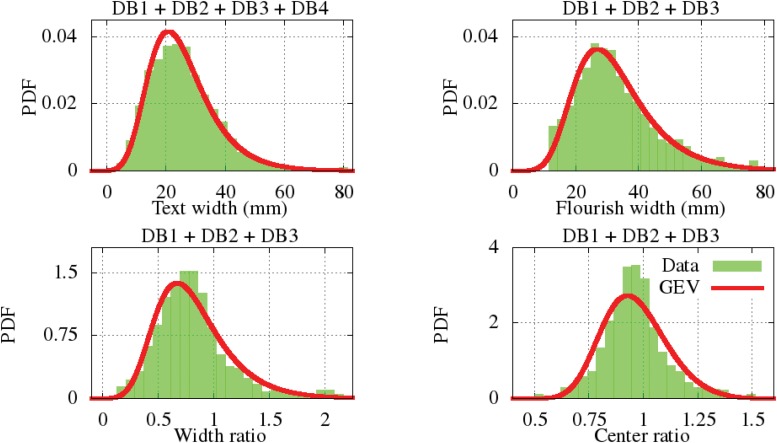
Text and flourish PDF relations approached by GEV.

In many cases, the text is inserted within or surrounded by a flourish. A relationship exists between the text and flourish width and geometric center of each. The text and flourish width and the ratio between these widths and the distance between the center of the text and flourish have been measured. These latter two aspect ratios locate the relative position of the text and flourish inside the signature envelope. The four distributions are depicted in Fig 13 with their GEV parameters presented at Table 3. The Kolmogorov-Smirnov test indicates that the signatures with text and flourish share similar text-flourish dependence in DB1, DB2 and DB3. However, because the DB4 dataset only includes a few signatures with a flourish, we have not included their data in these analyses. On average, the flourish width is slightly larger than the text width, which is normally around 25 mm, despite the larger space available for collecting the signatures. Such a small difference explains that the width ratio is near to one. Also we can deduce that both text and flourish appear centered on average, since the PDF maximum is near to one.

Two additional relations between the text and the flourish have been addressed: the temporal order in which they were written and the connection between them.

**Fig 14:**
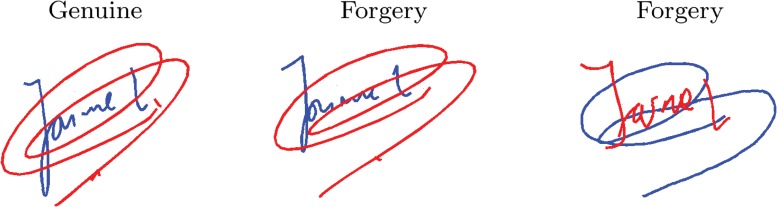
Forged signatures with text and flourish written in the same and different order than the genuine one. The blue line refers to the initial part of the signature and the red line the remainder: (left) genuine specimen where the name precedes the flourish; (center) and (right) represents forged signatures correctly and incorrectly drawn respectively.

Regarding the temporal order, it is noted that in the case of text plus only one flourish, 15.0% of the flourishes are written before the text in DB1; 8.1% in DB2; and 10.6% for DB3. No such data was available in the dataset DB4. Such an order generates a source of confusion for forgers because they usually imitate the signature image without information on the dynamics. As an example, Fig 14 shows a signature drawn in red. Note that the initial part of the signature is highlighted in blue. The forger sees an original image of the signature and then tries to reproduce it. Note that the forged signature in the center keeps the correct order but not the one to the right.

**Fig 15:**
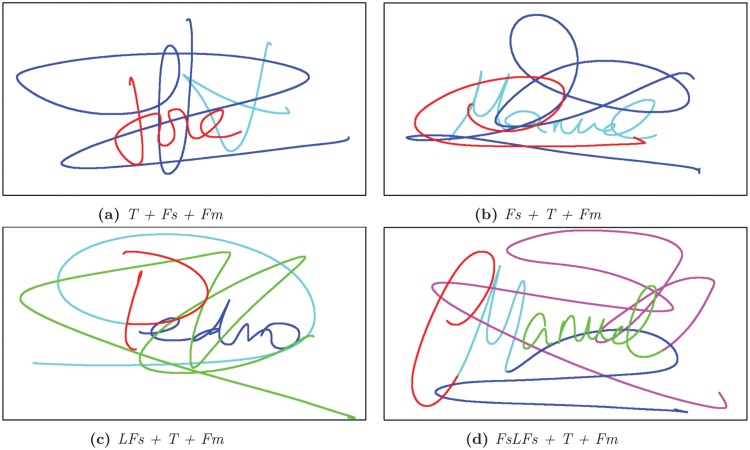
Probability of the different text-flourish structures. The colors represent the order in which the signature was written. From initial to final signature, the color order is defined as follows: red, cyan, blue, green and magenta.

We noted that complex structures are found in the databases when there is a text and a flourish. As stated above, we found many cases where signatures have associated flourishes. The 79%, 91.9%, 89.4% and 100% of the signatures in the datasets DB1, DB2, DB3 and DB4 respectively have a simple structure: they are composed of text plus one single flourish. Such flourishes sometimes appear connected to the text and we have found that 58% of users connect them. On the other hand, the rest of these signatures have a complex structure of the text plus two flourishes. The combination of the text and two flourishes allows us to define four cases: *i*) text plus two flourishes represented as *T+Fs+Fm*; *ii*) a secondary flourish followed by the text and the main flourish, *Fs+T+Fm*; *iii*) the initial text connected with the secondary flourish, *LFs+T+Fm* and; *iv*) the initial capital letter of the name enclosed by two secondary flourishes followed by the rest of the text and the main flourish *FsLFs+T+Fm*. Table 2 shows the probability distribution of these different structures in all analyzed datasets. Additionally, the Fig 15 depicts an example of each of these structures. We note that the more common complex structure is due to a graphically generated initial plus the rest of the text followed by a flourish. This is closely similar to signatures with simple structures, highlighting that Western signatures usually avoid excessive complexity.

**Table 2:** Relationship between the text and the flourishes in the complex structures for the Western databases.

**Cases**	**DB1**	**DB2**	**DB3**	**DB4**
T + Fs + Fm	7%	0%	5%	0%
Fs + T + Fm	22%	27%	37%	0%
LFs + T + Fm	68%	37%	47%	0%
FsLFs + T + Fm	3%	36%	11%	0%

The probability density functions previously represented for each selected feature can be analyzed. We have obtained parameters from each generalized extreme value approximation. Apart from the generalized extreme values, Table 3 shows the mean and variance of each function, the maximum probable value of the functions, the skewness and kurtorsis, which mainly interprets the function shape, the minimum and maximum values of the GEV and, finally, the mean square error estimator which measures the average of the squares of the errors between real values presented as a histogram and the measured function. These statistical parameters may be useful in further studies on the lexical morphology of signatures.

**Table 3:** Analytical results from Generalized Extreme Value distributions.

	**Shape *ξ***	**Scale *σ***	**Local. *μ***	**Mean**	**Variance**	**Pr. max**	**Skew.**	**Kurt.**	**min.**	**max.**	**MSE**
**Letters line 1. DB1-DB2**	-0.30	2.47	5.22	6.07	6.01	0.16	-0.06	2.71	1	12	4.12e-03
**Letters line 1. DB3-DB4**	-0.21	1.69	4.72	5.40	3.12	0.22	0.21	2.84	1	10	3.76e-03
**Letters line 2-1. DB1**	0.13	1.53	5.77	6.88	5.75	0.24	2.22	13.65	4	12	6.78e-03
**Letters line 2-2. DB1**	-0.34	2.15	4.09	4.77	4.34	0.18	-0.19	2.75	1	9	5.75e-03
**Slant All DBs**	-0.31	14.20	75.74	80.47	194.70	0.03	-0.11	2.72	12.31	120.12	4.84e-04
**Skew All DBs**	-0.09	7.78	3.81	7.69	81.75	0.05	0.70	3.75	-20.12	40.32	1.01e-03
**Corners (Fm) DB1-DB3**	-0.08	1.54	3.60	4.37	3.24	0.24	0.73	3.81	1	1	3.29e-03
**Corners (Fm) DB4**	-0.09	0.81	1.93	2.32	0.87	0.46	0.67	3.65	1	4	3.48e-02
**Corners (Fs) DB1**	0.50	1.20	1.83	3.68	∞	0.34	75.70	39520.80	1	7	1.55e-02
**Corners (Fs) DB2**	-0.06	0.71	2.21	2.57	0.71	0.52	0.80	4.04	1	5	1.38e-02
**Corners (Fs) DB3**	-0.33	0.55	2.02	2.20	0.28	0.72	-0.16	2.73	1	3	4.10e-03
**Text width All DBs**	-0.01	8.84	20.75	25.75	124.64	0.04	1.06	5.09	1.52	80.23	3.36e-04
**Flo. width DB1-DB3**	0.01	10.13	27.03	33.01	174.96	0.04	1.22	5.86	12.21	78.12	4.21e-04
**Width ratio DB1-DB3**	-0.02	0.27	0.67	0.82	0.11	1.38	1.06	4.95	0.13	2.30	2.24e-02
**Center ratio DB1-DB3**	-0.17	0.14	0.90	0.96	0.02	2.73	0.37	3.03	0.50	1.50	6.99e-02

We have studied lexical morphological variability through this paper: i.e. the differences between the parameters which define the particular lexical morphology of a Western signature. Moreover, these signatures have been collected following specific protocols in different laboratories. Therefore, the signatures have similar lexical morphological variability. As a future line of research, it might be desirable to study signatures captured in real scenarios like a bank or a supermarket. It is possible that in such environments we could find greater variability, for example, changes in the number of flourishes or flourish corners, changes in the number of letters and so on.

## Discussion

Any study of the lexical morphology of handwritten signatures embraces a number of disciplines because of the many factors that influence human signature design and appearance. Therefore, this study of lexical morphology modeling may be applied in several different areas.

In the biometric community, a relatively recent development is the synthesis of written signatures, on the basis of modeling the cognitive and neuromotor processes. Some such techniques are focused on the duplication of existing identities [54–60]. Other strategies call for the generation of completely new identities [35–37]. This latter approach requires: signature design, cognitive map modeling, neuromuscular motor simulation and, for off-line signature generation, an ink deposition model for generating realistic signature images. Lexical morphology modeling is required for the signature design stage. Thus text line morphology is one of the features which produces the text distribution.

It is often found that important historical documents are not complete or are damaged. This means that researchers have to cope with interpreting missing or degraded material [61–63]. Sometimes, incomplete handwriting signatures can be discovered in these documents. The counted signature parameters described in this paper could be considered as an additional tool in working with such documents.

Indexing is a further application where a model of lexical morphology can help significantly. The indexing consists in optimizing the speed and performance to find the required signature by matching a search query. Often the index design incorporates interdisciplinary concepts from linguistics, cognitive psychology, mathematics, informatics, physics, and computer science. Thus, the lexical morphology can for a quicker indexed search help to establish the relevant tree branch.

To support coherent decision making, it is useful in psychology and forensic science to know the normality or the weirdness of a given signature. In this context, a Bayesian paradigm has been accepted because of its capability of combining the personal judgment of a probability and the Forensic Expert’s evaluation of observed evidence (*E*) [64].

The Bayesian approach in its odds form assumes two hypotheses for some piece of evidence (*E*). Let *H*_1_ denote that the suspect has made the forged signature and let *H*_2_ denote that the suspect is not guilty and someone else has made the signature. Using the notation described in [64], the posterior odds is written in the form of the formula below. The first ratio corresponds to the priori probability ratio, the second is the likelihood ratio and the resultant is the posterior probability ratio. The priori probability ratio refers to the probability that a signature is written by the guilty versus the probability of such a signature being made by another person when background knowledge (*I*) is regarded as given or previously taken into account. The second part of the formula is the likelihood ratio of the probability of the observation if hypothesis 1 is true and the observation if hypothesis 2 is true. The posterior probability ratio represents the updated probability of the hypotheses given the actual observations.

$$\begin{array}{c} \frac{Pr(H_{1}|I)}{Pr(H_{2}|I)}\times \frac{Pr(E|I,H_{1})}{Pr(E|I,H_{2})}=\frac{Pr(H_{1}|E,I)}{Pr(H_{2}|E,I)} \end{array}$$ (5)

Our work can contribute to the forensics in the evaluation of the likelihood ratio. This ratio measures the relative strength of support for evidence (*E*) against the hypothesis 1 and its alternative. The evidence in respect to the alternative hypothesis is a value corresponding to the occurrences of the observation in a pool of signatures. This value is the product of the probabilities calculated during the lexical and morphological analysis of the parameters of the questioned signature.

**Fig 16:**
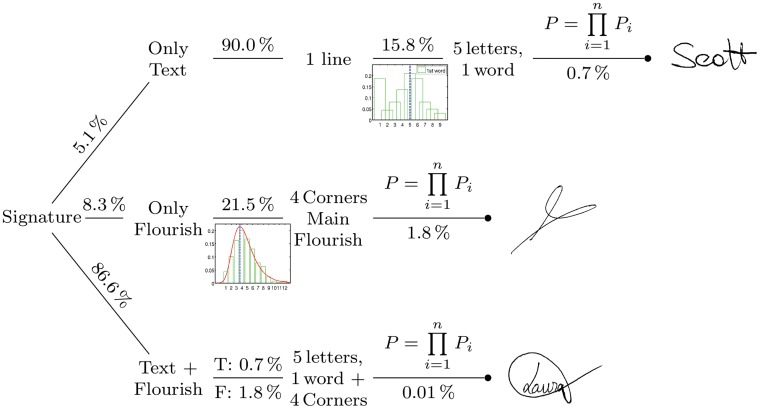
Example of simplified probability tree for the DB1.

We have assumed here that Western signatures follow a pattern according to a common lexical morphology. This study reports the probability values of the lexical morphology of written signatures through probability density functions. As such, we can determine the frequency of the more typical signature types: only text, only flourish or both text and flourish. Using the obtained probabilities, it is possible to draw a probability tree for the datasets. As an example, a simplified probability tree for the DB1 is shown in Fig 16. It indicates the more usual signatures found in the datasets. For instance, we can see that a signature with text and flourish is the most typical. The most probable signature has a lexical morphology similar to the signature drawn by the end of each branch. Note that this feature has been calculated using signatures produced only by healthy and relatively young donors.

## Conclusion

This paper studies the lexical morphology of Western style handwritten signatures. From a large set of possible parameters, we have selected a small set in order to gain a better understanding of the main factors which characterize the way the signatures are performed. We use various statistical distributions. For each parameter we have calculated the Probability Density Function using Generalized Extreme Value functions. Each selected feature has been validated using signatures from five real Western public databases: MCYT, GPDS960GRAYsignature, NISDCC, SUSIG and SVC corpuses.

The characterized parameters are presented and are presumed helpful for addressing the normality of signatures in general. Certainly, human behavior is rather difficult to measure in this field, as in others. However, this statistical analysis attempts to bring closer the knowledge of the behavior of the lexical morphology of signatures for a human population.
